# The beta-secretase-derived C-terminal fragment of APP (C99): a key determinant for intraneuronal pathology in the 3xTgAD mouse

**DOI:** 10.1186/1750-1326-8-S1-P63

**Published:** 2013-10-04

**Authors:** Inger Lauritzen, Raphaelle Pardossi-Piquard, Charlotte Druon, Elizabeth Brigham, Daniel Abraham, Sébatien Ranaldi, Linda Chami, Julie Dunys, Fréderic Checler

**Affiliations:** 1 IPMC-CNRS UMR7275, Sophia Antipolis, France; 2 Elan Pharmaceuticals, San Fransico, USA; 3 Cap Delta - Parc Euromédecine, Montpellier, France

## Background

Triple-transgenic mice (3xTgAD) overexpressing Swedish-mutated b-amyloid-precursor-protein (APP_swe_), P310L-Tau (Tau_P301L_) and physiological levels of M146V-presenilin-1 (PS1_M146V_) display extracellular amyloid-beta peptides (Abeta) deposits and Tau tangles at late ages. These mice also show an early, age-dependent and hippocampus specific accumulation of intra-neuronal APP-related materiel, which was firstly reported to correspond to Abeta. However, more recent work has disputed the presence of intra-neuronal Abeta and questioned its role in synaptic pathology and behavior.

## Material and methods

In the present work we used multiple approaches (biochemical, immunohistological, genetic and pharmacological) to determine the exact nature of the intracellularly accumulating catabolite in the 3xTgAD. The 3xTgAD and nonTg were provided by Dr.LaFerla and the 2xTgAD mice were obtained by firstly crossing 3xTgAD and nonTg mice and then inter-crossing the F1 progeny. One group of mice was treated by oral gavage with the gamma-secretase inhibitor (ELND006, Elan Pharm.) during 2 weeks.

## Results

Here we identify the beta-secretase-derived C-terminal fragment of APP (C99) as the earliest APP catabolite and main contributor to the intracellular APP-related immunoreactivity in 3xTgAD mice. C99 is detected as early as about 3 months of age which is several months before intracellular Abeta can be detected. C99 accumulation occurs mainly in the CA1/subicular interchange area of the hippocampus corresponding to the first region exhibiting plaques and tangles in old mice. In other regions such as the cortex, C99 does not accumulate with age, although APP is expressed to similar levels. We show that C99 accumulation is not linked to a defective gamma-secretase processing. First of all, gamma-secretase activity is identical in the hippocampus and the cortex. Second of all, mice lacking the FAD PS1 mutant knock-in (2xTgAD) similarly accumulate C99 indicating that the accumulation is not triggered by the PS1 mutation. Finally, pharmacological inhibition of the gamma-secretase rapidly and importantly raises the levels of C99 in young mice.

## Conclusions

Altogether, our work identifies C99 as the first and main APP catabolite accumulating intra-cellularly in the 3xTgAD mouse suggesting its role as a key initiator and determinant for intra-neuronal pathology in the 3xTgAD mouse.

